# Micronucleus formation in human tumour cells: lack of correlation with radiosensitivity.

**DOI:** 10.1038/bjc.1993.17

**Published:** 1993-01

**Authors:** C. Bush, T. J. McMillan

**Affiliations:** Radiotherapy Research Unit, Institute of Cancer Research, Sutton, Surrey, UK.

## Abstract

The micronucleus (MN) test has been carefully characterized in four human tumour cell lines of widely differing radiosensitivity. Two radioresistant bladder carcinoma cell lines (MGH-U1 and RT112), one sensitive medulloblastoma cell line (D283MED) and a sensitive neuroblastoma cell line (HX142) were used. The number of MN per Gy of ionising radiation was 0.13 for HX142, 0.17 for D283MED, 0.21 for RT112 and 0.26 for MGH-U1. This does not rank the cell lines in the same order of radiosensitivity as clonogenic cell survival where the surviving fraction at 2 Gy (SF2) was 0.11 for HX142, 0.2 for D283MED, 0.62 for RT112 and 0.53 for MGH-U1. This discrepancy between MN formation and cell death leaves doubt as to the potential usefulness of the MN test as a rapid assay of radiosensitivity but it has potential implications for the mechanistic basis of radiosensitivity in these cells.


Br. J. Cancer (1993), 67, 102–106 © Macmillan Press Ltd., 1993

### Micronucleus formation in human tumour cells: lack of correlation with radiosensitivity

C. Bush & T.J. McMillan

*Radiotherapy Research Unit, The Institute of Cancer Research, Cotswold Road, Sutton, Surrey SM2 5NG, UK.*

**Summary** The micronucleus (MN) test has been carefully characterised in four human tumour cell lines of widely differing radiosensitivity. Two radioresistant bladder carcinoma cell lines (MGH-U1 and RT112), one sensitive medulloblastoma cell line (D283MED) and a sensitive neuroblasoma cell line (HX142) were used. The number of MN per Gy of ionising radiation was 0.13 for HX142, 0.17 for D283MED, 0.21 for RT112 and 0.26 for MGH-U1. This does not rank the cell lines in the same order of radiosensitivity as clonogenic cell survival where the surviving fraction at 2 Gy ($SF_2$) was 0.11 for HX142, 0.2 for D283MED, 0.62 for RT112 and 0.53 for MGH-U1. This discrepancy between MN formation and cell death leaves doubt as to the potential usefulness of the MN test as a rapid assay of radiosensitivity but it has potential implications for the mechanistic basis of radiosensitivity in these cells.

Micronuclei (MN) are formed when chromosomal fragments behave independently of the remaining chromosomes during the division of damaged cells. The presence of MN is thus considered to reflect genotoxic damage. The MN test has been applied to studies of environmental and industrial hazards (Heddle *et al.*, 1983; Heddle *et al.*, 1990; Meng & Zhang, 1990). The relationship between genotoxicity, as measured by the MN test and cytotoxic damage following ionising radiation treatment has been investigated by a number of authors and a general correlation has usually been found between MN formation and slow growth or the absence of colony formation (Joshi *et al.*, 1982; Stap & Aten, 1990). Also MN frequency changes in proportion to cell kill when a given cell type is irradiated under various conditions (Midander & Revesz, 1980).

Due to its simplicity there has been much interest in exploiting this relationship between MN frequency and cytotoxicity to provide a rapid test of radiosensitivity in human cells. In particular the use of the MN test as a predictive test of human tumour cell radiosensitivity is being explored (Peters *et al.*, 1986; Streffer *et al.*, 1986). Fenech and Morley (1985, 1986) established the cytokinesis-block method as the simplest and most precise application of the MN test. The blocking of cytokinesis by the use of Cytochalasin-B enables identification of cells in their first post-treatment mitotic division by the production of binucleate cells (BNC). This overcomes one of the major cell kinetic problems which plague the micronucleus test.

A complication of the MN test in non-diploid cells was identified by Revell and his colleagues (Revell, 1983). Fragment loss in a pure diploid Syrian hamster cell line, as detected by MN formation, correlated on a 1:1 basis with inhibited growth. However, a spontaneous tetraploid variant required on average 2 MN per lethal event while a hypotetraploid variant required $>2$ MN per lethal event. Thus in non-diploid cell lines the direct correlation between MN formation and cell death did not hold: it appeared that such cells could tolerate fragment loss to a greater degree than the diploid line.

Since a large proportion of human tumour cells are aneuploid this complication could be a significant factor. In the present study we have therefore studied four human tumour cell lines of widely differing radiosensitivity in order to investigate the relationship between cell kill, as assessed by a clonogenic assay, and micronucleus formation.

Correspondence: T.J. McMillan.
Received 9 January 1992; and in revised form 4 September 1992.

### Materials and methods

### *Cell lines*

Four human tumour cell lines were used: HX142 is a neuroblastoma cell line which was originally established from xenografted tumour tissue (Deacon *et al.*, 1985); RT112 and MGHU1 are both bladder carcinoma cell lines (Kato *et al.*, 1977; Masters *et al.*, 1986); D283MED is a medulloblastoma cell line (Friedman *et al.*, 1985) and was kindly provided by Dr D. Bigner. All cell lines were grown in monolayer in Ham's F12 medium with 10% foetal bovine serum, $100\,mg\,l^{-1}$ streptomycin and $10^5$ units $l^{-1}$ penicillin.

Table I shows the modal chromosome numbers and the cell cycle distributions of the four cell lines. The former have been taken from previous publications which include these cell lines. The cell cycle distributions are mean values taken from two or three experiments with each cell line. Despite attempts to standardise the state of growth of the cells at the time of analysis these results were rather variable. However, there is a tendency for RT112 and MGH-U1 to have a lower $G_1$ fraction and a higher S-phase fraction than the other two cell lines. These cell cycle distributions were measured 24 h after plating when cells were growing well. This is directly comparable to the timing of irradiation of cultures for MN experiments. These distributions are also likely to be accurate for the survival experiments since these were prepared from exponentially growing cultures.

### *Micronucleus test*

Single-cell suspensions (0.4-1 × $10^4$ cells/plate) were plated into 3.5 cm plastic Petri dishes containing a 15 mm plastic coverslip (Thermanox). These were incubated at 37°C in a gassing incubator (3% $O_2$, 5% $CO_2$, 92% $N_2$) for 24 h; (48 h, for HX142) to allow the cells to establish a monolayer before irradiation. The plating density was chosen to prevent

**Table I**

| *Cell line* | *Modal chromosome number*[a] | *Cycle cell distribution*[b] $G_1$ | *S* | $G_2/M$ |
|---|---|---|---|---|
| HX142 | 46 | 71 (65–83) | 18 (9–25) | 11 (8–20) |
| D283MED | 47 | 72 (69–74) | 21 (17–25) | 7 (6–9) |
| MGH-U1 | 47 | 45 (36–51) | 45 (40–53) | 10 (9–11) |
| RT112 | 46 | 41 (40–42) | 45 (40–50) | 14 (10–18) |

[a]Value taken from previous publications (Bigner *et al.*, 1988; McMillan *et al.*, 1990). [b]Analysed by flow cytometry. Values given are means of a minimum of two independent measurements with the range of values given in parentheses. Cells were stained using propidium iodide and analysed using an Ortho Cytofluoragraf 50M as described by Yang *et al.* (1990).

confluence being achieved during the course of the experiments. Irradiations were carried out in a portable perspex incubator at 37°C using a 1100 Ci (41 TBq) $^{60}Co$ source. Two hours after irradiation the cells were fed with fresh medium containing 1 μg ml$^{-1}$ Cytochalasin-B (Sigma). After incubating for 1–6 days in the presence of Cytochalasin-B, the dishes were fixed with 70% ethanol and stained with 3% Giemsa. The coverslips were then mounted on glass microscope slides. A minimum of 100 BNC were scored per slide using a Zeiss light microscope under oil (× 320). The number of MN per BNC was recorded. A minimum of two (in some cases six) independent experiments were performed for each cell line in each stage of the characterisation and final analysis of the MN test.

**Figure 1** Clonogenic cell survival curves for the four cell lines (from McMillan *et al.*, 1990, and Powell *et al.*, 1992).

*Preparation of Cytochalasin B*

Cytochalasin-B was dissolved in dimenthylsulphoxide and stored in aliquots at − 70°C at a concentration of 1 mg ml$^{-1}$. Freshly thawed stock solution was diluted in Ham's F12 medium containing 10% foetal calf serum (Imperial Laboratories) to produce the required concentration.

*Criteria for identification of micronuclei*

Only MN within binucleate cells were included and in addition they conformed to the following criteria: (1) they were separate from the main nuclei; (2) they were no larger than one-third the volume of the main nuclei; (3) their morphology and staining properties were similar to those of the main nuclear material.

### Results

*Clonogenic survival*

The radiosensitivity of the four cell lines as measured by clonogenic assay have been published elsewhere (McMillan *et al.*, 1990; Holmes *et al.*, 1990; Powell *et al.*, 1992). For reference these survival curves are reproduced in Figure 1. The surviving fractions at 2 Gy ($SF_2$) are 0.1 for HX142, 0.18 for D283MED, 0.53 for MGH-U1 and 0.62 for RT112. This range of sensitivities is representative of the spectrum of $SF_2$ values seen in a larger number of human tumour cell lines (Steel *et al.*, 1989).

*Cytochalasin-B treatment*

Cytochalasin-B is toxic at high doses. The growth of all four cell lines was affected to a similar degree by the presence of Cytochalasin-B and it was found that a 24 h treatment with Cytochalasin-B (1 μg ml$^{-1}$) reduced survival of RT112 and MGH-U1 to 70% and 40% respectively in a clonogenic assay (data not shown). It was therefore important to find a dose which provided a satisfactory block in cytokinesis without causing too much cell kill.

**Figure 2** Relationship of MN frequency to concentration of Cytochalasin-B. Cells were fixed 3 days after irradiation. Points are means and standard errors.

**Figure 3** Effect of time after irradiation on MN frequency. Points are means and standard errors.

Figure 2 shows the effect of Cytochalasin-B on MN frequency at 3 days. In the absence of Cytochalasin-B counts were made in all cells rather than just BNC and in this situation levels of MN per cell were extremely low in all cases: 0.03 per cell for MGH-U1, 0.02 for RT112, 0.003 for D283MED and 0.02 for HX142. As expected, these levels were generally lower than those obtained in the presence of Cytochalasin-B due to the dilution effect of the separation of daughter nuclei into separate cells. A plateau of MN frequency is reached at 0.5 $\mu g ml^{-1}$ for MGH-U1, D283MED and HX142, and at 1.0 $\mu g ml^{-1}$ for RT112. We selected 1.0 $\mu g ml^{-1}$ Cytochalasin-B for all the cell lines, which led to 60–80% of cells being binucleate on day 3 for MGH-U1, HX142 and RT112 but a lower number of BNC (30%) for D283MED.

*Time of MN expression*

Sufficient time is needed after irradiation to allow cells to progress through one nuclear division in order to display MN formation. Figure 3 shows the expression of MN as a function of time in the presence of 1 $\mu g ml^{-1}$ Cytochalasin-B. For MGH-U1 and RT112 the MN values levelled out after 2 days. For D283MED, a plateau of MN frequency was

**Figure 4** Radiation dose-response curves for all four cell lines. Points are means and standard errors. Data were fitted using at least squares linear regression analysis.

**Figure 5** Relationship of lethal lesions (– ln(SF) read from curves in Figure 1) to MN frequency (from data points in Figure 4). Data were fitted using linear regression analysis. ○ = RT112, ● = MGH-U1, ■ = HX142, □ = D283MED.

reached by 3 days. For HX142 of MN seemed to continue rising slightly up to day 5. By days 5 and 6 the number of multinucleate cells increased markedly in all cell lines which severely complicates analysis. It was therefore considered that day 3 was a satisfactory time at which to measure the expression of micronuclei.

*Micronucleus formation*

For each of the four cell lines the number of MN per BNC increased with dose of ionising radiation (Figure 4). The increase is approximately linear in each case, although for RT112 there is a suggestion of an upward curvature. Assuming linearity, the MN induction values were 0.10 per Gy for HX142, 0.11 per Gy for D283MED, 0.23 per Gy for RT112 and 0.21 per Gy for MGH-U1. Contrary to our expectation the two most sensitive lines (HX142 and D283MED) had fewer MN at any given dose than the two resistant lines. This difference also exists when the proportion of cells with MN is plotted against dose (not shown) rather than MN per cell.

*Relationship between cell kill and MN formation*

Figure 5 shows the MN per BNC against the number of lethal lesions for the range of doses used. For this the number of lethal lesions was calculated as – ln (SF) from the curves in Figure 1. The relationship is not the same for all cell lines. For RT112 and MGH-U1 approximately 1 MN corresponded to 1.4 lethal events. However, for HX142 and D283MED the presence of one MN per BNC translated to 12.8 and 7.0 lethal lesions respectively, if the lines in Figure 5 were extrapolated.

### Discussion

This study has examined the relationship between the incidence of micronuclei in human tumour cells with clonogenic cell survival in order to evaluate the possible use of the MN test as a predictive test of tumour cell radiosensitivity. Despite careful attention to the parameters of the MN test, including cytochalasin dose and timing of analysis, it was found that the MN test did not rank the cell lines in the same order of sensitivity as was done with clonogenic cell survival. Thus, a straightforward interpretation of these results is that the MN test is not a useful predictive test.

Breakdown in a direct relationship between cell death and MN formation has been detected previously in other contexts. Geard and Chan (1990), for example, found the MN frequency relationship to be different for a given level of cell kill when cells were irradiated at high and low dose rates. Wandl *et al.* (1989) found a good linear relationship between survival and MN frequency in a series of human renal cell carcinomas examined soon after removal from the patient, but this relationship was not the same for each tumour. Also van Beuningen *et al.* (1981) found that hyperthermia increased MN formation without altering clonogenic cell survival.

As well as the implications for predictive testing the finding of a lack of a simple relationship between MN frequency and cell kill is of interest mechanistically. In their studies on Syrian hamster cells, Revell (1983) found that any drift away from a pure diploid cell line upset the relationship between MN and inhibited growth, therefore it is perhaps not surprising that the exponentially growing human tumour cells do not conform to a 1:1 relationship. The explanation for this could be partly practical and partly biological.

One feature of the Revell studies was they were performed on cells irradiated while synchronised in $G_1$. This has the value that any chromosome fragment formed will be reflected in a genetic loss from both daughter cells at the first cell division. When cells are irradiated post-S-phase, however, it is *chromatid* fragments which are formed. Thus only one of the daughter cells may be affected. A chromosome fragment loss would therefore be reflected in a reduced colony formation whereas chromatid fragment loss would not decrease colony number, it would merely make the colonies one division behind the control colonies. It is not clear how S-phase cells would behave in this regard, although chromatid-type aberrations outnumber chromosome-type aberrations in cells irradiated in S-phase. Due to the asynchronous growth of the cell lines in the present study and the apparent differences in the cell cycle distributions we must consider what effect this would have on our results.

First of all the lack of synchronisation in all the cell lines would lead to MN having a reduced effect on colony formation as the damaged $G_2$/M cells (and perhaps at least some of those in S-phase) would still form colonies if only one daughter cell was affected. This would lead to a MN:lethal lesion ratio greater than 1. In each case here this ratio is less than 1. The sensitive cell lines (HX142 and D283MED) do have more cells in $G_1$ for which each MN is more likely to contain chromosomal fragments which would then reduce colony formation. However, the scale of the differences in MN per lethal lesion between the cell lines is unlikely to be fully explained by these differences in cell cycle distribution.

The influence of ploidy should also be considered as Revell (1983) found that cells with more chromosomes suffered increased damage but they also exhibited greater tolerance of that damage. The four cell lines in this study all have very similar modal chromosome numbers, therefore ploidy would seem to be unimportant. Nevertheless, the cell lines do not have normal karyotypes, so that perhaps karyotypic instability or functional hemizygosity are influencing these results.

The conclusion from the data with the sensitive lines is that MN loss in the first division after irradiation is not a large contributor to the death in these cells. At the extreme this may suggest that they are dying from a non-mitotic death. Apoptosis has recently been reported to be a cause of death in gamma-irradiated cells (Stephens *et al.*, 1991) but we have found that although apoptosis can be detected in D283MED this is not a significant effect in these cells (Ung & McMillan, in preparation). Alternatively, these cell lines may be inefficient at converting chromosome fragments into MN or MN might only be expressed in later divisions after irradiation, i.e. the fragment exclusion probability at the first and subsequent divisions may differ between the cell lines. If this factor did differ between our cell lines we would have perhaps expected the distributions of MN per cell to differ for a given average number of MN per cell. Our ability to examine this is restricted due to the finding that the highest MN frequency detected in HX142 and D283MED is low for the dose range studied. However, in our analysis of the distributions of the number of MN per cell (data not shown) we have been unable to detect differences between the cell lines. A detailed analaysis of the cytogenetic changes induced by radiation in these cells is needed to investigate these possibilities.

Overall the data presented suggest that the MN test may not be a satisfactory rapid test of the radiosensitivity of human tumour cells, although it remains to be fully tested within a single tumour type. However, it may provide useful information regarding the way different cells handle radiation-induced damage.

We are grateful to Professor G.G. Steel and Mr J.H. Peacock for their advice throughout this project and to Dr D. Bigner for providing the D283MED cell line. Also we are indebted to Mrs S. Stockbridge and Miss R. Couch for the careful preparation of this manuscript. This study was supported by the Cancer Research Campaign and the Medical Research Council.

**Results**

BIGNER, S.H., MARK, J., FRIEDMAN, H.S., BIEGEL, J.A. & BIGNER, D.D. (1988). Structural chromosomal abnormalities in human medulloblastoma. *Cancer Genet. Cytogenet.*, **30**, 91-101.

DEACON, J.M., WILSON, P.A. & PECKHAM, M.J. (1985). The radiobiology of human neuroblastoma. *Radiother. Oncol.*, **3**, 201-209.

FENECH, M. & MORLEY, A. (1985). Solutions to the kinetic problem in the micronucleus assay. *Cytob.*, **43**, 233-246.

FENECH, M. & MORELEY, A.A. (1986). Cytokinesis-block micronucleus method in human lymphocytes: effect of *in vivo* ageing and low dose X-irradiation. *Mut. Res.*, **161**, 193-198.

FRIEDMAN, H.S., BURGER, P.C., BIGNER, S.C., TROJANOWSKI, J., HALPERIN, E.C. & BIGNER, D.D. (1985). Establishment and characterisation of the human medulloblastoma cell line and transplantable xenograft D283 MED. *J. Neuropathol. Exp. Neurol.*, **44**, 592-605.

GEARD, C.R. & CHEN, C.Y. (1990). Micronuclei and clonogenicity following low- and high-dose-rate gamma-irradiation of normal human fibroblasts. *Radiat. Res.*, **124**, S56-S61.

HEDDLE, J.A., BOUCH, A., KAHN, M.A. & GINGERICH, J.D. (1990). Concurrent detection of gene mutations and chromosomal aberrations induced *in vivo* in somatic cells. *Mutagen.*, **5**, 179-184.

HEDDLE, J.A., HITE, M., KIRKHART, B., MAVOURNIN, K., MACGREGOR, J.T., NEWELL, G.W. & SALAMONE, M.F. (1983). The induction of micronuclei as a measure of genotoxicity. *Mut. Res.*, **123**, 61-118.

HOLMES, A., MCMILLAN, T.J., PEACOCK, J.H. & STEEL, G.G. (1990). The radiation dose-rate effect in two human neuroblastoma cell lines. *Br. J. Cancer*, **62**, 791-795.

JOSHI, G.P., NELSON, W.J., REVELL, S.H. & SHAW, C.A. (1982). Discrimination of slow growth from non-survival among small colonies of diploid Syrian hamster cells after chromosome damage induced by a range of X-ray doses. *Int. J. Radiat. Biol.*, **42**, 283-296.

KATO, T., IRWIN, R.J. & PROUT, G.R. (1990). Cell cycles in two cell lines of human bladder carcinoma. *Int. J. Radiat. Biol.*, **58**, 427-438.

MASTERS, J.R.W., HEPBURN, P.J., WALKER, L., HIGHMAN, W.J., TREJDOSIEWICZ, L.K., POVEY, S., PARKAR, M., HILL, B.T. & RIDDLE, P.R. (1986). Tissue culture models of transitional cell carcinoma: characterization of 22 human urothelial cell lines. *Cancer Res.*, **46**, 3630-3636.

MCMILLAN, T.J., CASSONI, A.M., EDWARDS, S., HOLMES, A & PEACOCK, J.H. (1990). The relationship of DNA double-strand break induction to radiosensitivity in human tumour cell lines. *Int. J. Radiat. Biol.*, **58**, 427-438.

MENG, Z. & ZHANG, L. (1990). Observation of frequencies of lymphocytes with micronuclei in human peripheral blood cultures from workers in a sulphuric acid factory. *Environ. Molec. Mutagen.*, **15**, 218-220.

MIDANDER, J. & REVESZ, L. (1980). The frequency of micronuclei as a measure of cell survival in irradiated cell populations. *Int. J. Radiat. Biol.*, **38**, 237-242.

PETERS, L.J., BROCK, W.A., JOHNSON, T., MEYN, R.E., TOFILON, P.J. & MILAS, L. (1986). Potential methods for predicting tumor radiocurability. *Int. J. Radiation Oncol. Biol. Phys.*, **12**, 459-467.

POWELL, S.N., MCMILLAN, T.J. & STEEL, G.G. (1992). *In vitro* radiosensitivity of human medulloblastoma cell lines. *J. Neuro-Oncol.* (in press).

REVELL, S.H. (1983). Relationships between chromosome damage and cell death. In: *Radiation-Induced Chromosome Damage in Man*, New York: Alan R. Liss, Inc., p.215-233.

STAP, J. & ATEN, J.A. (1990). Comparison of radiation sensitivity for three cell lines as measured by the cloning assay and the micronucleus test. *Strahlenther. Onkol.*, **166**, 761-763.

STEEL, G.G., MCMILLAN, T.J. & PEACOCK, J.H. (1989). The picture has changed in the 1980s. *Int. J. Radiat. Biol.*, **56**, 525-537.

STEPHENS, L.C., ANG, K.K., SCHULTHEISS, T.E., MILAS, L. & MEYN, R.E. (1991). Apoptosis in irradiated murine tumors. *Radiat. Res.*, **127**, 308-316.

STREFFER, C., VAN BEUNINGEN, D., GROSS, E., SCHABRONATH, J., EIGLER, F.-W. & REBMANN, R. (1986). Predictive assays for the therapy of rectum carcinoma. *Radiother. Oncol.*, **5**, 303-310.

VAN BEUNINGEN, D., STREFFER, C. & BERTHOLDT, G. (1981). Mikronukleusbildung im vergleich zur uberlebensrate von menschlichen melanmzellen nach röntgen-, neutronenbestrahlung und hyperthermie. *Strahlentherapie*, **157**, 600-606.

WANDL, E.O., ONOS, K., KAIN, R., HERBSTHOFER, T., HIENERT, G. & HOBARTH, K. (1989). Linear correlation between surviving fraction and the micronucleus frequency. *Int. J. Radiat. Biol.*, **56**, 771-775.

YANG, X., DARLING, J.L., MCMILLAN, T.J., PEACOCK, J.H. & STEEL, G.G. (1990). Radiosensitivity, recovery and dose-rate effect in three human glioma cell lines. *Radiother. Oncol.*, **19**, 49-56.


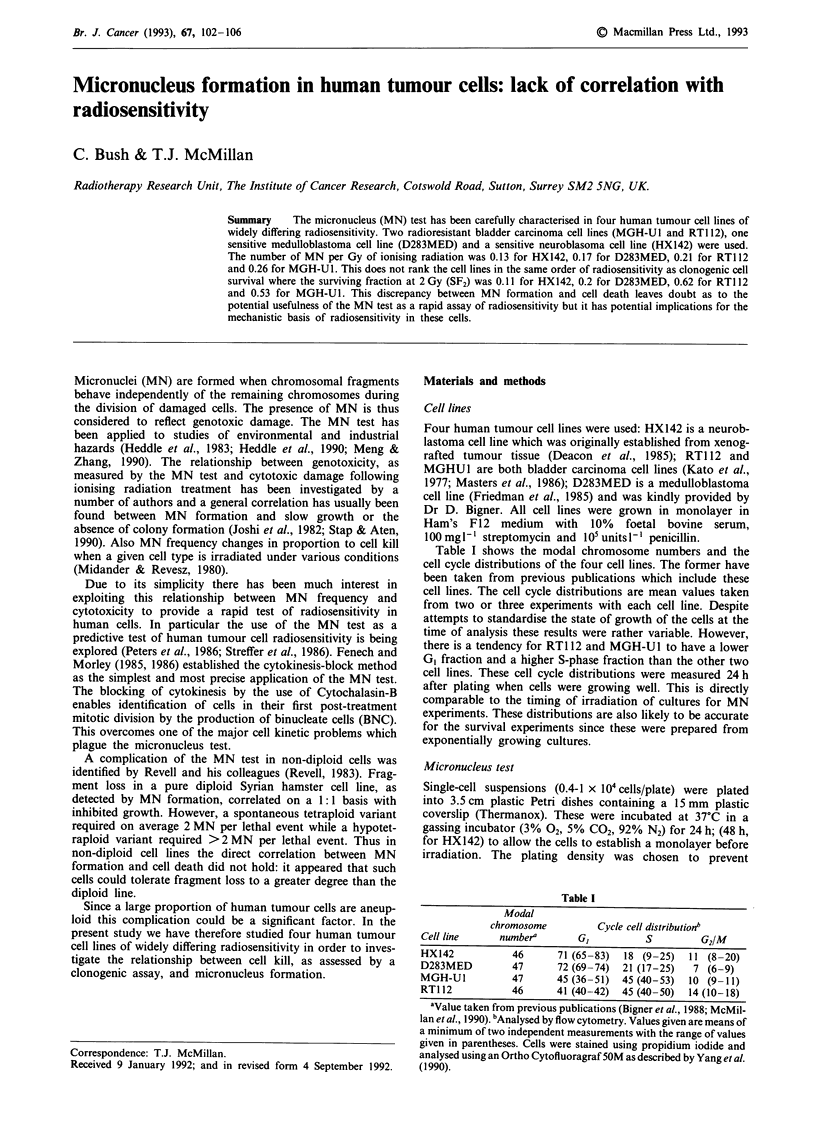

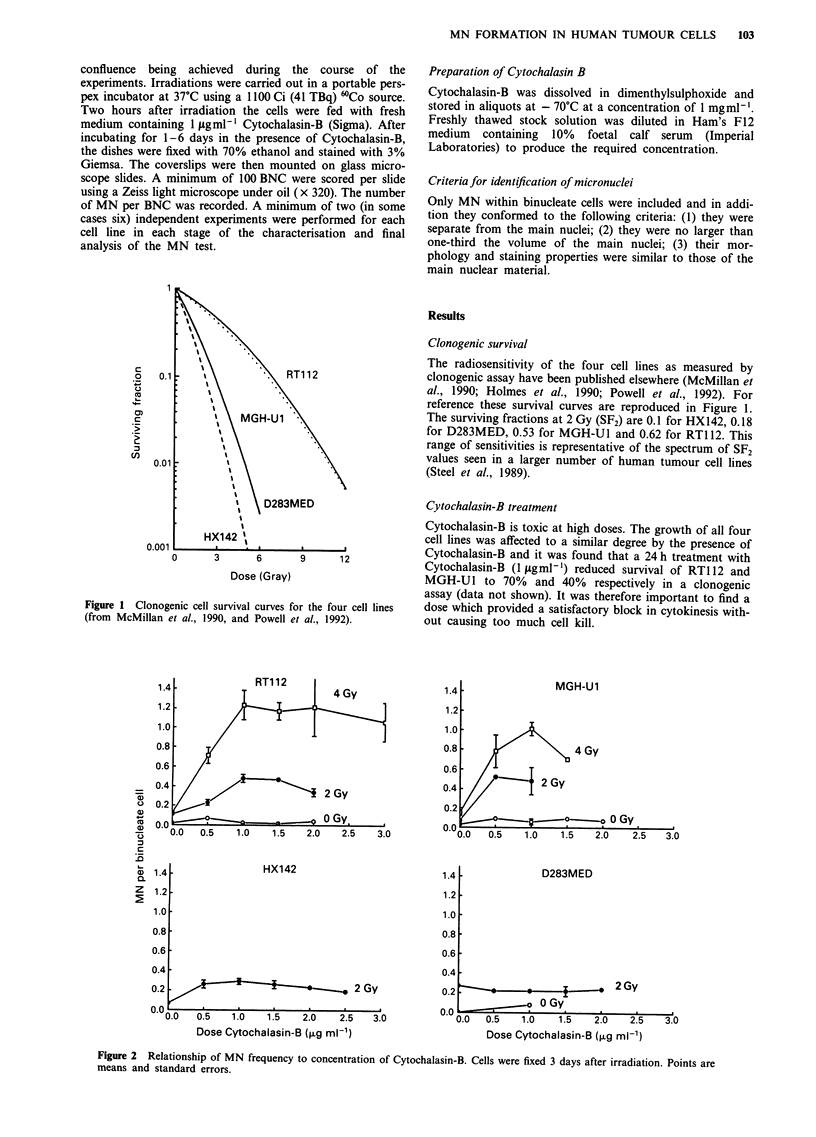

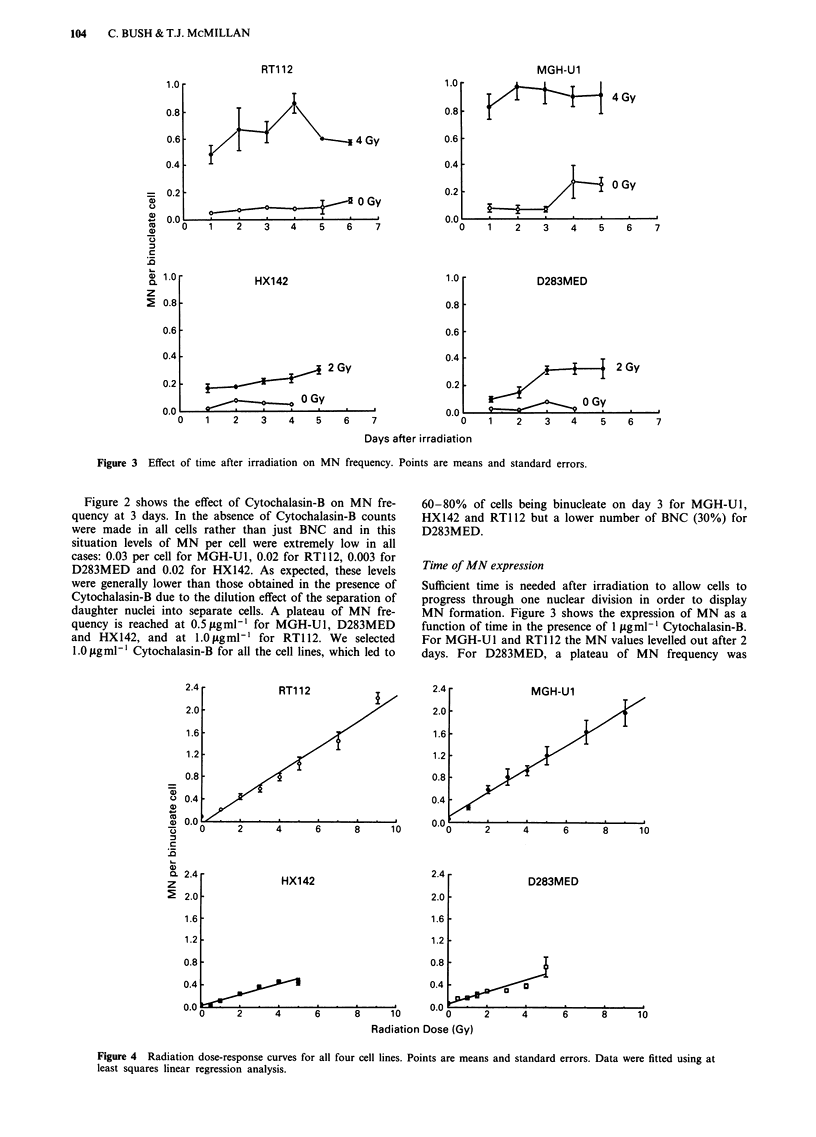

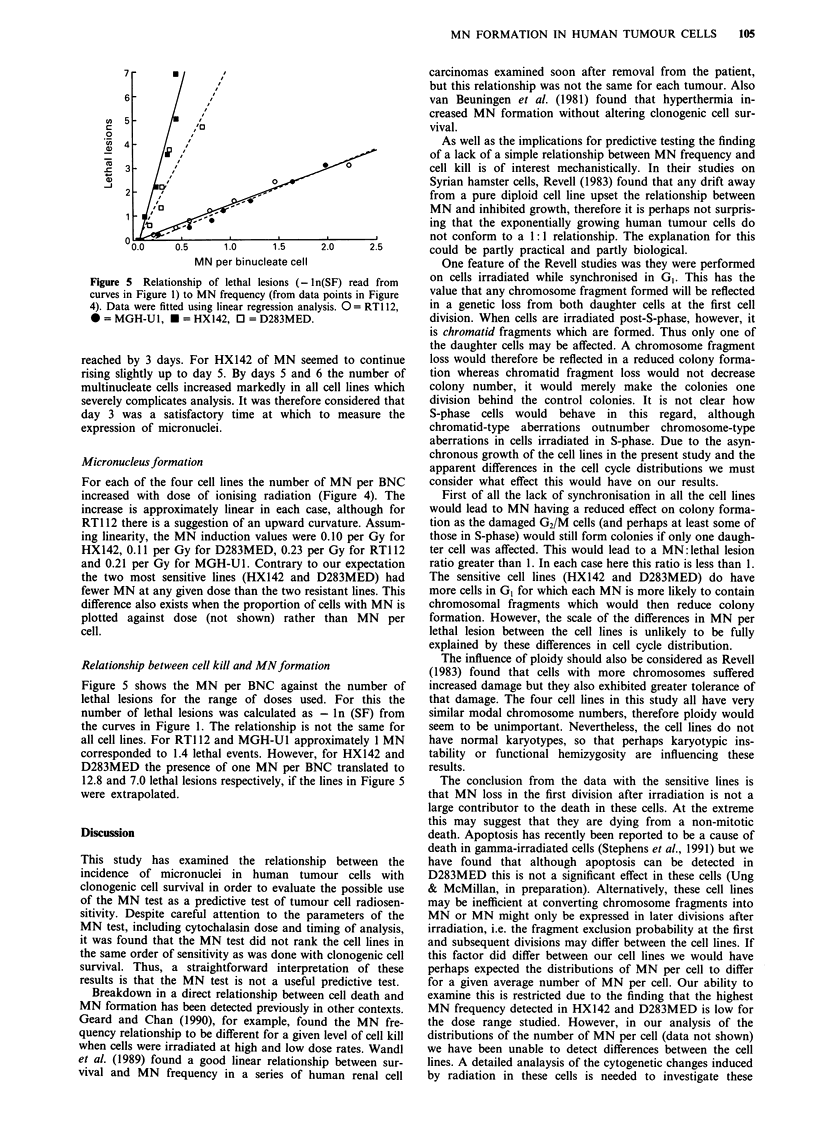

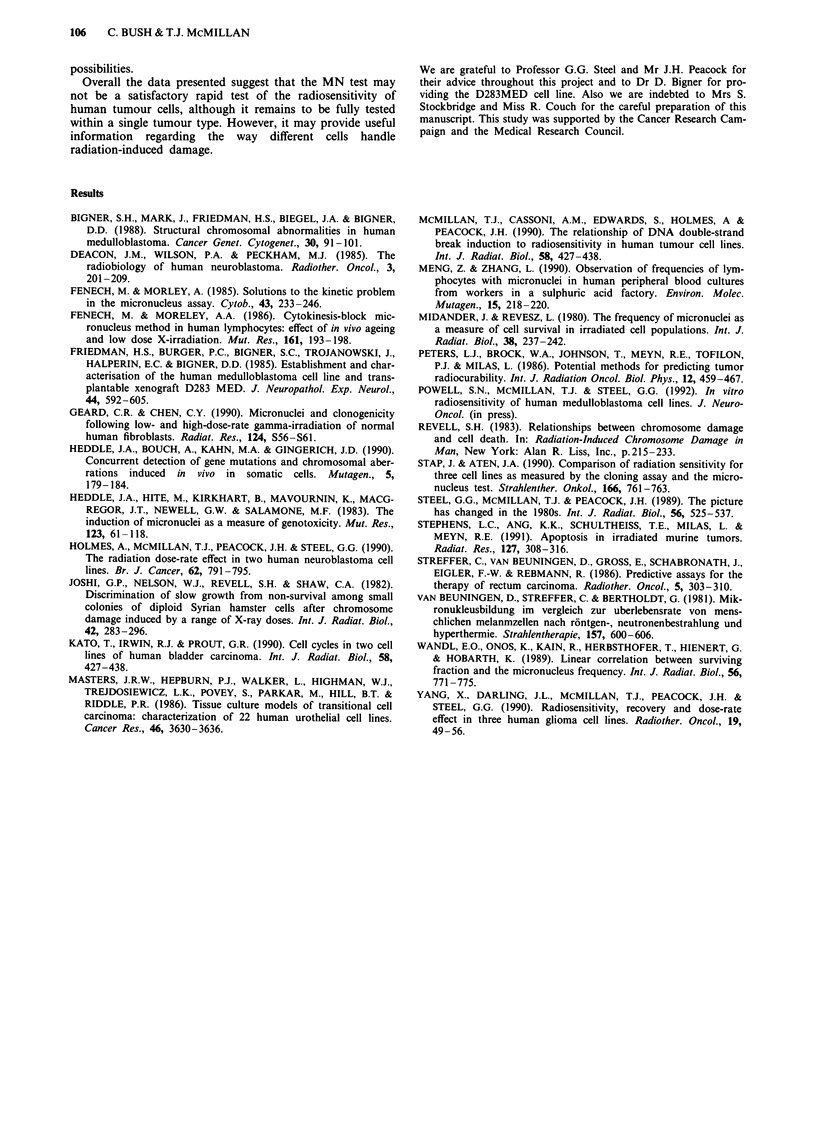


## References

[OCR_00666] Bigner S. H., Mark J., Friedman H. S., Biegel J. A., Bigner D. D. (1988). Structural chromosomal abnormalities in human medulloblastoma.. Cancer Genet Cytogenet.

[OCR_00671] Deacon J. M., Wilson P. A., Peckham M. J. (1985). The radiobiology of human neuroblastoma.. Radiother Oncol.

[OCR_00680] Fenech M., Morley A. A. (1986). Cytokinesis-block micronucleus method in human lymphocytes: effect of in vivo ageing and low dose X-irradiation.. Mutat Res.

[OCR_00676] Fenech M., Morley A. (1985). Solutions to the kinetic problem in the micronucleus assay.. Cytobios.

[OCR_00685] Friedman H. S., Burger P. C., Bigner S. H., Trojanowski J. Q., Wikstrand C. J., Halperin E. C., Bigner D. D. (1985). Establishment and characterization of the human medulloblastoma cell line and transplantable xenograft D283 Med.. J Neuropathol Exp Neurol.

[OCR_00692] Geard C. R., Chen C. Y. (1990). Micronuclei and clonogenicity following low- and high-dose-rate gamma irradiation of normal human fibroblasts.. Radiat Res.

[OCR_00697] Heddle J. A., Bouch A., Khan M. A., Gingerich J. D. (1990). Concurrent detection of gene mutations and chromosomal aberrations induced in vivo in somatic cells.. Mutagenesis.

[OCR_00705] Heddle J. A., Hite M., Kirkhart B., Mavournin K., MacGregor J. T., Newell G. W., Salamone M. F. (1983). The induction of micronuclei as a measure of genotoxicity. A report of the U.S. Environmental Protection Agency Gene-Tox Program.. Mutat Res.

[OCR_00709] Holmes A., McMillan T. J., Peacock J. H., Steel G. G. (1990). The radiation dose-rate effect in two human neuroblastoma cell lines.. Br J Cancer.

[OCR_00714] Joshi G. P., Nelson W. J., Revell S. H., Shaw C. A. (1982). Discrimination of slow growth from non-survival among small colonies of diploid Syrian hamster cells after chromosome damage induced by a range of x-ray doses.. Int J Radiat Biol Relat Stud Phys Chem Med.

[OCR_00726] Masters J. R., Hepburn P. J., Walker L., Highman W. J., Trejdosiewicz L. K., Povey S., Parkar M., Hill B. T., Riddle P. R., Franks L. M. (1986). Tissue culture model of transitional cell carcinoma: characterization of twenty-two human urothelial cell lines.. Cancer Res.

[OCR_00721] McMillan T. J., Cassoni A. M., Edwards S., Holmes A., Peacock J. H. (1990). The relationship of DNA double-strand break induction to radiosensitivity in human tumour cell lines.. Int J Radiat Biol.

[OCR_00733] McMillan T. J., Cassoni A. M., Edwards S., Holmes A., Peacock J. H. (1990). The relationship of DNA double-strand break induction to radiosensitivity in human tumour cell lines.. Int J Radiat Biol.

[OCR_00739] Meng Z. Q., Zhang L. Z. (1990). Observation of frequencies of lymphocytes with micronuclei in human peripheral blood cultures from workers in a sulphuric acid factory.. Environ Mol Mutagen.

[OCR_00745] Midander J., Révész L. (1980). The frequency of micronuclei as a measure of cell survival in irradiated cell populations.. Int J Radiat Biol Relat Stud Phys Chem Med.

[OCR_00750] Peters L. J., Brock W. A., Johnson T., Meyn R. E., Tofilon P. J., Milas L. (1986). Potential methods for predicting tumor radiocurability.. Int J Radiat Oncol Biol Phys.

[OCR_00764] Stap J., Aten J. A. (1990). Comparison of radiation sensitivity for three cell lines as measured by the cloning assay and the micro-nucleus test.. Strahlenther Onkol.

[OCR_00769] Steel G. G., McMillan T. J., Peacock J. H. (1989). The radiobiology of human cells and tissues. In vitro radiosensitivity. The picture has changed in the 1980s.. Int J Radiat Biol.

[OCR_00773] Stephens L. C., Ang K. K., Schultheiss T. E., Milas L., Meyn R. E. (1991). Apoptosis in irradiated murine tumors.. Radiat Res.

[OCR_00778] Streffer C., van Beuningen D., Gross E., Schabronath J., Eigler F. W., Rebmann A. (1986). Predictive assays for the therapy of rectum carcinoma.. Radiother Oncol.

[OCR_00789] Wandl E. O., Ono K., Kain R., Herbsthofer T., Hienert G., Höbarth K. (1989). Linear correlation between surviving fraction and the micronucleus frequency.. Int J Radiat Biol.

[OCR_00795] Yang X., Darling J. L., McMillan T. J., Peacock J. H., Steel G. G. (1990). Radiosensitivity, recovery and dose-rate effect in three human glioma cell lines.. Radiother Oncol.

[OCR_00783] van Beuningen D., Streffer C., Bertholdt G. (1981). Mikronukleusbildung im Vergleich zur Uberlebensrate von menschlichen Melanomzellen nach Röntgen-, Neutronenbestrahlung und Hyperthermie.. Strahlentherapie.

